# Prompting and Modeling of Coping Strategies during Childbirth

**DOI:** 10.1007/s40617-023-00837-6

**Published:** 2023-09-13

**Authors:** Ashley Greenwald

**Affiliations:** University of Nevada Reno, 1664 North Virginia Street, Reno, NV 89557 USA

**Keywords:** Behavior analysis, Behavioral medicine, Childbirth, Software

## Abstract

There is ample evidence to suggest that upright positions and mobility during labor improve birth outcome, including shorter duration of childbirth and reduced risk of cesarean section. The use of nonpharmacological interventions for pain management during childbirth are recommended by major health-care institutions and medical providers, however, the current methodologies for training coping strategies for use during labor have not shown to be effective on mobility or birth outcome. The purpose of this study was to apply an in-vivo teaching technology to the current childbirth model to prompt an imitative repertoire of empirically demonstrated labor coping strategies. Results of this study concluded that the introduction of a software using immediate prompting and video modeling increased the frequency and variability of labor behaviors during unmedicated labor for birthing persons and their partners.

Childbirth is a natural physiological event that the average woman of childbearing age and ability in the United States will experience approximately twice in her lifetime. In the United States, most birth occurs in a hospital setting with use of pharmaceuticals for pain relief or for shortening the duration of labor, often resulting in the need for increased medical intervention to mitigate side effects (Clark et al., 2008; Kuklina et al., 2009). Over 80% of people use an epidural during labor, however, almost 50% of people who do not want epidurals during labor end up receiving one (Goer & Romano, 2012). This information suggests that the preferences of laboring persons and the current childbirth supports available may be misaligned.

It is common medical practice for a person to remain in a relatively stationary and in a supine position during labor and childbirth (Goer & Romano, 2012), yet lack of ambulation and fixed position during labor may not promote ideal birth outcomes. A Cochrane review (Lawrence et al., 2013) analyzed 25 randomized controlled trials involving over 5,000 participants and concluded the first stage of labor was approximately 1 hr and 20 min shorter in duration for people who were ambulatory during labor. Other positive outcomes of movement during labor demonstrated in this review included a reduction in the risk of cesarean birth, lower rates of epidural analgesia use, and reduced neonatal intensive care unit admissions. It is recommended that birthing persons be encouraged and supported to use upright and mobile positions of their choice during early and active labor (Lawrence et al., 2013). In addition, in a report summarizing best practices in perinatal care, appropriate methods for pain relief are based in behavior and movement: “Avoid the use of medications in labor. Preferably pain management should use nonpharmacological methods, such as ambulation, changing positions, massage, relaxation, breathing, acupuncture, and others. Avoid epidural analgesia as a routine method of pain management” (Chalmers et al., 2001, p. 206). An additional large meta-analysis of nonpharmacological approaches for pain management concluded that when compared to routine medical interventions, nonpharmacological approaches were found to have better birth outcomes for mothers and babies (Chaillet et al., 2014).

Widely acknowledged coping behaviors for labor are often taught through childbirth education courses. Information received during childbirth education varies by course and methodology but often includes explanation of the birth process, medical interventions available, and use of coping skills for pain management including various birthing positions, relaxation strategies, and comfort measures (i.e., Bilgin et al., 2020, Chaillet et al., 2014). Childbirth education has demonstrated to be effective in building perception of birth confidence (Howarth & Swain, 2019), knowledge of delivery (Pinar et al., 2018), and use of problem-solving skills during labor (Ucar & Golbasi, 2018). Literature on childbirth education and birth outcome is more limited; however, recent research demonstrated there is no effect on the type of delivery methods used or obstetrical birth outcome because of participation in childbirth education (Bilgin et al., 2020). In addition, socioeconomic factors influence access to childbirth education, as birth preparation classes are most likely to be accessed by people who are white, highly educated, and privately insured (Sperlick et al., 2019), thereby indicating the need to expand access to educational supports and resources.

Although the World Health Organization (2015) recommended the use of nonpharmacological interventions for pain management during childbirth, the current methodologies for training coping strategies for labor have not shown to influence birth outcomes (Bilgin et al., 2020). Considering childbirth education from a science of behavior, limited training without demonstration of proficiency is likely to result in poor skill acquisition. In addition, even if some skills are acquired, those skills may not generalize to a new and highly stressful environment, resulting in a skill deficit for both the birthing person and their partner during labor. Although not widely studied, this skill deficit has been documented in the literature (e.g., Spiby et al., 1999) however, little research has been done to address it. In one sizeable survey on labor support, 91% of people found immersion in water, heat/cold packs, or use of a birth ball to be somewhat or very helpful in managing labor pain, however, only 7% of people used these strategies (Declercq et al., 2007). Although labor strategies are empirically based and widely taught, the likelihood of engaging in them during labor is low.

Decades of research have specifically mentioned the need for more effective prompting methodologies of nonpharmacological coping strategies during childbirth (e.g., Escott et al., 2009; Slade et al., 2000; Spiby et al., 1999). Behaviors such as movement, relaxation, and partner support promote a more natural progression of labor and research demonstrated that these variables result in a lower risk of cesarean section (Caton et al., 2002; Lawrence et al., 2013) and increased satisfaction with birth outcome (Green & Hotelling, 2014). The World Health Organization’s Safe Childbirth Checklist includes the presence of a birth companion to provide coaching and guidance (World Health Organization, 2015), yet partners are often underprepared and professional labor support persons, or doulas, are only used by a small percentage of the population (Declercq et al., 2007).

To address the previously mentioned barriers to use of coping strategies during labor, a behavioral software program called StorkAssist was created to provide a treatment package inclusive of empirically demonstrated labor coping strategies. StorkAssist includes two main behavioral interventions: in-vivo prompting and video modeling of labor support strategies. Prompting is an intervention wherein a stimulus is added to the environment, signaling the correct behavior to engage in to access reinforcement. Modeling an intervention that demonstrates an exact skill to the learner and can be done live or with a video model. Instructions and modeling are the primary components of behavioral skills training and have significant implications for increasing skill acquisition (e.g., Ward-Horner & Sturmey, 2012). The purpose of this study was to examine and understand how a behavioral software treatment package, including momentary instruction through prompting and modeling of labor behaviors, might increase the frequency and variability of evidence-based coping strategies engaged in by laboring persons and their partners during unmedicated labor.

## Method

### Participants and Setting

Six dyads, including a laboring participant and one labor support partner, each, were recruited for this study, for a total of 12 people. People of childbearing age and all educational, ethnic, and socioeconomic backgrounds were eligible to participate and no knowledge or experience with childbirth was required for participation. People that were considered to have a high-risk pregnancy and/or could not labor outside of a hospital setting for a time-period were excluded. In addition, people that had hired a doula, or professional labor support coach, were excluded, as the work of the doula would interfere with the current investigation.

All six of the laboring persons included in this study identified as female and were between the ages of 27 and 42 years old. A brief survey of birth experience was given to each participant prior to the experimental phase, and it identified that four of the participants were primipara, meaning it was their first birth, and two of the participants were multipara, meaning they had previously given birth before. All six of the partners identified as male with ages unknown. Five of the laboring persons identified as Caucasian and one Asian, with one also identifying as Hispanic. Five of the support partners identified as Caucasian and one as Asian.

All participants had a healthy and term pregnancy, were considered low risk by their medical providers, and had received medical guidance to allow their labor to begin at home. Therefore, the baseline and experimental portions of the study took place in the participants’ homes prior to the time in which their medical provider required that they depart for the hospital to deliver their baby.

### Materials

**Fig. 1:**
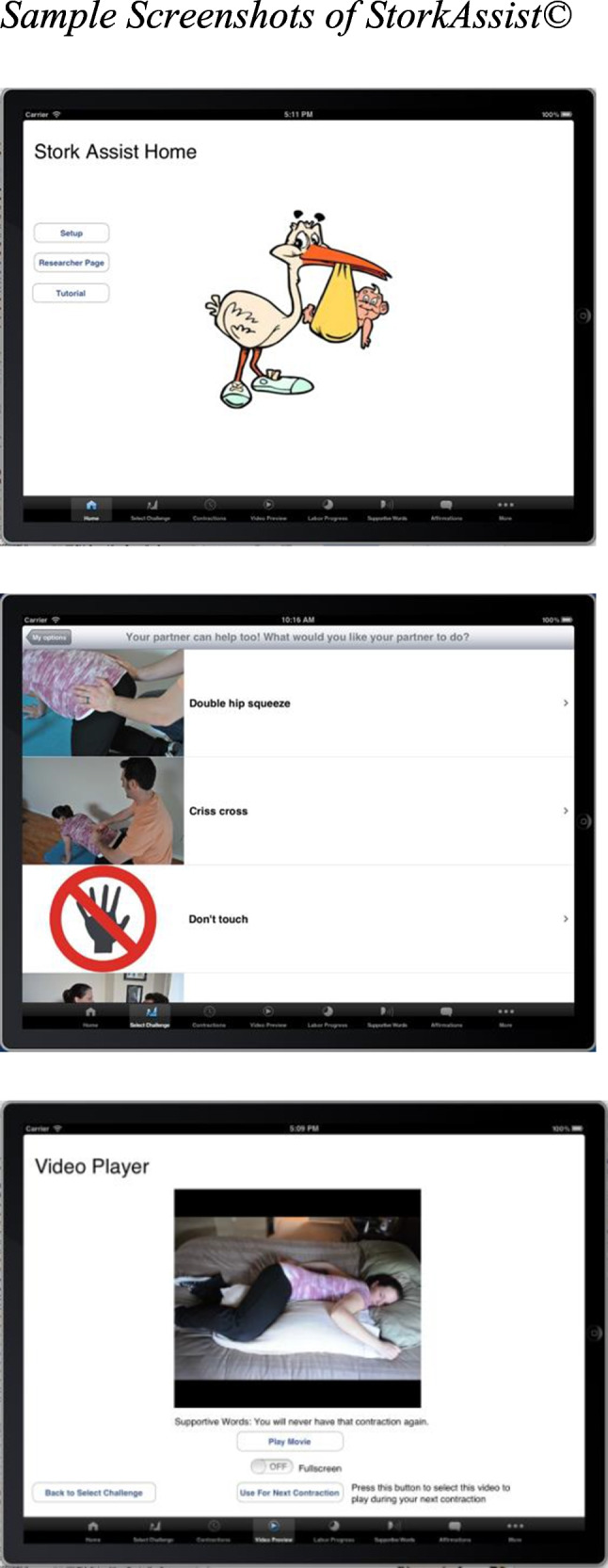
Sample Screenshots of StorkAssist. *Note*. Top Panel: The welcome screen of the software application depicts the ability to navigate to the set-up screen, the brief tutorial, or the various program tabs. Middle Panel: Example of a list of support options for the partner to choose from that appears once the laboring person has previously selected their challenge, preferred position, and preferred behavior. Bottom Panel: Video preview tab shows the user the unique combination of previously selected labor coping behaviors for the laboring person and partner that will play during a contraction

This study was conducted using the StorkAssist software program, a behavioral application on labor support specifically designed for this research (see Fig. 1). StorkAssist relies on the use of antecedent stimuli that evoke imitative behavior using prompting and video modeling to demonstrate effective behavioral coping techniques. Each laboring person and their partner were provided with an iPad with the StorkAssist software installed. StorkAssist incorporates evidence-based labor support and comfort measures including movement, relaxation, breathing, massage, and positions while providing prompts and models for the laboring person and their partner to imitate.^1^ The software was programmed to collect information about the momentary and unique needs of the laboring person and provide options for one to six pain management behaviors for the person to imitate followed by one to six support person behaviors for their partner to imitate. Below is a description of each feature of the software.

### Tutorial

When the participants opened the software, they were taken through a brief tutorial on how to use the program. Screen shots of the most relevant pages were used to highlight certain features with explanations of how to use each feature. The tutorial is eight total pages in length and the participants were required to click through each page before the program began. The participants could navigate back to the tutorial at any time by pressing the tutorial button on the homepage.

### Set-Up

Immediately following the tutorial, the participants were prompted to input as much information as they could regarding: (1) the three medically measurable areas of labor progress (station, dilation, effacement); (2) amenities and resources available to them during labor (birth ball, massage tools, shower, bathtub, heat, and cold packs); and (3) special conditions (fetal monitors, epidural, or confined to bed). Given that the experimental portion of the current evaluation occurred outside of a medical setting, many of these special conditions only applied to the participants if they choose to continue using the software following the experimental condition and upon entering the hospital. Based on the conditions entered in the set-up screen, the program automatically filtered out coping strategy options that are irrelevant or would not be beneficial.

### Home Screen

The home screen (see Fig. 1) is where the participants were directed after completing the set-up menu. The home screen consisted of three buttons and eight tabs (the 8^th^ tab generated two additional tabs): Home, Set-up, Researcher Page, Tutorial, Select Challenge, Contractions, Video Preview, Supportive Words, Affirmations, Breathing, and Massage. The eight tabs were on every screen of the software for navigation purposes. The *Set-Up* button directed the user back to the set-up screen so that at any time during their labor, the participants could make changes to the progress, amenities, and special conditions that they had available to them. The *Researcher Page* button was a password-protected button that allowed the researcher to make changes to the backend of the system from the device. The researcher could: (1) set the time intervals for the reminder pop-ups that could be turned on/off by the participant; (2) change the intervals in which the user was asked to update their labor progress; and (3) turn on/off the questions to the user following each contraction. The *Tutorial* button navigated the user back to the first screen of the tutorial so they could review it at any time. Each one of the tabs at the bottom of the home screen navigated the user to a different feature of the program, the most relevant of which are explained below.

### Select Challenge

The *Select Challenge* tab is the main feature of the program where the participant could select a common labor challenge that they are presently facing (e.g., fear/stress/panic, exhaustion, baby in bad position, pain, progressing too quickly, not progressing). The pain selection is the only selection that guided the user to an additional menu screen in which they could select the location of the pain (stomach, hip/thigh, back). After selecting the challenge, the participant was presented with general position options that correlated with the challenge selected and any special conditions established previously in the set-up menu. The participant was then prompted to select a general position (sitting, standing, laying, kneeling, squatting) that would be most comfortable or convenient for them. Based on their input from the setup screen and from the challenges options, the program generated between two and six specific labor options for the user to select. Pictures and words appeared on the selection screen to ensure the specific coping strategy was understood. After the participant selected a behavior for themselves, the program identified up to six partner support behaviors that would complement the laboring person’s selection (see Fig. 1). Upon completion of their respective choices, a video preview of the combination of selections automatically appeared (see Fig. 1). The participants could choose to move this video to the queue to use with the next contraction, or they could go back to select different challenge support techniques.

### Contractions

The participants were instructed to navigate to the *Contractions* tab at the onset of a labor contraction. Upon pressing the *Start Contraction* button, the previously queued video began playing. If the participant had not yet queued up a video, a default 4 s breathing video played. At the end of the contraction, the participant selected the *Contraction End* button, which ended the video model. At this point, the participant could determine if they want to engage in the same set of coping strategies or navigate back to the *Select Challenge* tab to identify other effective coping strategies.

### Procedures

The researcher met with the participants one time prior to the onset of labor. This meeting took no more than 30 min with the primary goal of completing consent forms and answering research related questions. During this meeting, the participants were allowed to view the StorkAssist eight-slide tutorial on the iPad as part of the consent protocol. The time spent engaged with the iPad software prior to labor did not exceed 5 min. During this initial meeting, the participants also completed a brief survey of their prior labor experience and childbirth education. All participants including the pregnant person and their birth partner signed consent forms to participate and viewed the StorkAssist tutorial.

Participants were instructed to call the researcher at the onset of early labor defined as contractions 10 min apart for at least 1 hr. At this time, the researcher or research assistant^2^ arrived at the home of the participants and determined if they were in early or active labor, based on the pattern of contractions. Contractions that were 5 min apart or less were considered to be active labor whereas contractions more than 5 min apart were considered to be early labor. The above definitions are consistent with the obstetrical literature (Liao et al., 2005). All six dyads were observed during one baseline and one intervention session during unmedicated labor in a home or community setting of their choice.

To respect the private and intimate nature of birth, a total time interval was used so that participants could anticipate how long the experimental session would last. The total time that the researcher spent observing the participants across conditions was 2 hr and the participants were instructed that the researcher would not engage with them during this time. For experimental purposes, each individual contraction was identified as the interval of observation, so an interval-based metric was selected for the following experimental conditions. Baseline was conducted across a predetermined number of contractions (5–15 contractions) for each participant prior to receiving the iPad with the software intervention. During the intervals in baseline, the researcher used partial interval recording to monitor the labor behaviors of both the laboring person and their partner. The baseline phase was nonconcurrent for all participants, as each participant began their labor on a different day. The intervention phase began immediately following the predetermined number of baseline contractions. During the intervention phase, the participant was handed the iPad with the StorkAssist software on it and instructed to use it as much as they want. The participants were then able to engage with the software program while the researcher observed for the remainder of the 2 hr session. During the intervals in intervention, the researcher used the same partial interval recording methodology to monitor the labor behaviors of both the laboring person and their partner. Given the timed nature of the total observation (2 hr) and the variability of intervals within and between participants, the exact number of contractions in the intervention condition varied across dyads. When the experimental condition was complete, the participants were given the option to keep the iPad with the software for the remainder of their labor or return it to the researcher.

Following the birth, the researcher emailed the participants an anonymous postdelivery survey link, which included both social validity questions and birth outcome questions. Quantitative and qualitative social validity questions were asked including: (1) Did you find the program helpful during labor? (Yes/No); (2) Did your partner find the program helpful during labor? (Yes/No); (3) If you were to give birth again, would you use the program? (Yes/No); (4) Would you recommend the program to a friend? (Yes/No); (5) What features of the program were most helpful?; and (6) Provide any additional thoughts or comments. As part of the same postdelivery survey, birth outcome questions were asked on use of pain medications during labor (intravenous pain medication, epidural, or none) and method of delivery (vaginal, vaginal assisted, or cesarean section).

#### Experimental Design

Experimental data were examined using a nonconcurrent multiple baseline across participants design during early and active labor with the time interval being one contraction. There were two groups of three dyads each; one group of three dyads were monitored during early labor and one group of three dyads were monitored during active labor. Early and active labor definitions were consistent with medical literature and total session length was 2 hr.

The independent variable was the treatment package in software format (StorkAssist) consisting of visual prompting, choice-making, and video modeling of childbirth coping strategies. The main dependent variables were engagement in coping behaviors by both the laboring person and their partner, as well as variability in behaviors. Additional dependent variables measured included the option to keep the iPad software for the remainder of labor, use of pain medication while in the hospital, and birth outcome.

## Response Measurement, Interobserver Agreement, and Treatment Fidelity

A primary data collector observed all sessions and collected data on labor behaviors and partner behaviors during each session. All sessions were video recorded and a trained research assistant independently collected data from the videos for interobserver agreement purposes.

Labor behaviors were defined before beginning the current evaluation. Data were collected on productive and non-productive labor behaviors for the laboring person, although only productive labor behaviors (hereafter collectively referred to as labor behaviors) were presented in the following data sets, as nonproductive labor behaviors (i.e., screaming) were rarely observed. Labor behaviors included the position of the laboring person, their body movement, and their vocalizations. Positions for the laboring person included sit, stand, kneel, lay, and squat. Body movements included rhythmic sway, lean forward, rock on a ball or chair, flail, or no movement. Vocalizations included deep breaths, hum, yell, or none. Partner behaviors included partner movements and partner vocalizations. Partner behaviors were identified as counterpressure (defined as partner hands or object held by partner pressed firmly against the laboring person’s lower back), double hip squeeze (defined as two hands applying pressure to the laboring person’s hips), application of heat or cold packs, position support (defined as holding, steadying, or supporting the laboring person in the chosen position), massage/touch, or none. Partner vocalizations included supportive words of encouragement, explanation of the position or support strategy, or none.

During each session, the total numbers of labor and partner behaviors were recorded in each interval. An interval was defined as the self-report of the onset of the noticeable muscle tightening of the uterus throughout the duration of the contraction and ending upon the full release of the tightening. As the study was conducted in the home environment without access to medical equipment, the research team relied upon the self-report from the participant to determine the contraction start and end times. Labor behaviors during contractions were recorded and observed for 45 s in early labor and 60 s in active labor, intervals that are consistent with the medical average for contraction duration during each phase of labor (Liao et al., 2005).

A secondary dependent variable of variability, defined here as a change in response selection, was calculated by observing novel behaviors, or behaviors that were different from behaviors engaged in during the previous interval. For example, if sitting and rocking occurred in interval one and again in interval two, there were two novel behaviors in interval one and zero novel behaviors in interval two. As an alternative, if in interval three, standing occurred, there was one novel behavior scored in interval three. A behavior that was engaged in during interval two and then not observed again until interval seven would be coded as a novel behavior, thereby indicating behavioral variability.

Interobserver agreement was calculated using total interval agreement methodology in which all behaviors coded within an interval either agreed or did not. Agreement was defined as a point-to-point correspondence between the primary observer and the secondary observer. Agreements were divided by the total number of intervals per participant and multiplied by 100%. Interobserver agreement was calculated for 71.4% to 100% of intervals across participants. The quality and ability to capture the video with both the participant and the partner remaining in the frame dictated the number of intervals in which interobserver agreement data were scored, with the intention to score as many intervals as possible. The arithmetic mean agreement across all six dyads was 91.8% (range: 80%–98.5%). The arithmetic mean agreement was 94.8% for the laboring persons’ positions (range: 80%–100%), 77.8% for the laboring persons’ movements (range: 20%–100%), and 93.6% for the laboring persons’ vocalizations (range: 76.6%–100%). The arithmetic mean agreement was 96.7% for partner behavior (range: 80%–100%) and 96.1% for partner vocalizations (range: 85%–100%). The lower agreement scores (i.e., 20% for the laboring person’s movement) were due to poorer video quality and an inability to clearly determine more subtle movements via video.

Treatment fidelity, or use of the iPad application during the experimental session, was also accounted for. During the baseline sessions, no participants had access to the iPad. During experimental conditions, a data log of inputs into StorkAssist, including the selected behaviors for participants and partners, was captured by the iPad for each participant during the experimental condition and was able to be compared to the video feeds to determine if the participants were in fact using the software during the experimental session. Treatment fidelity was defined as either engagement or nonengagement with StorkAssist during the respective baseline and experimental intervals. Treatment fidelity data were collected for all participants with 100% of participants not interacting with the iPad during baseline intervals and, apart from the sleeping partner in Dyad #202, all active participants engaged with the software during 100% of intervals in the experimental condition.

## Results

**Fig. 2:**
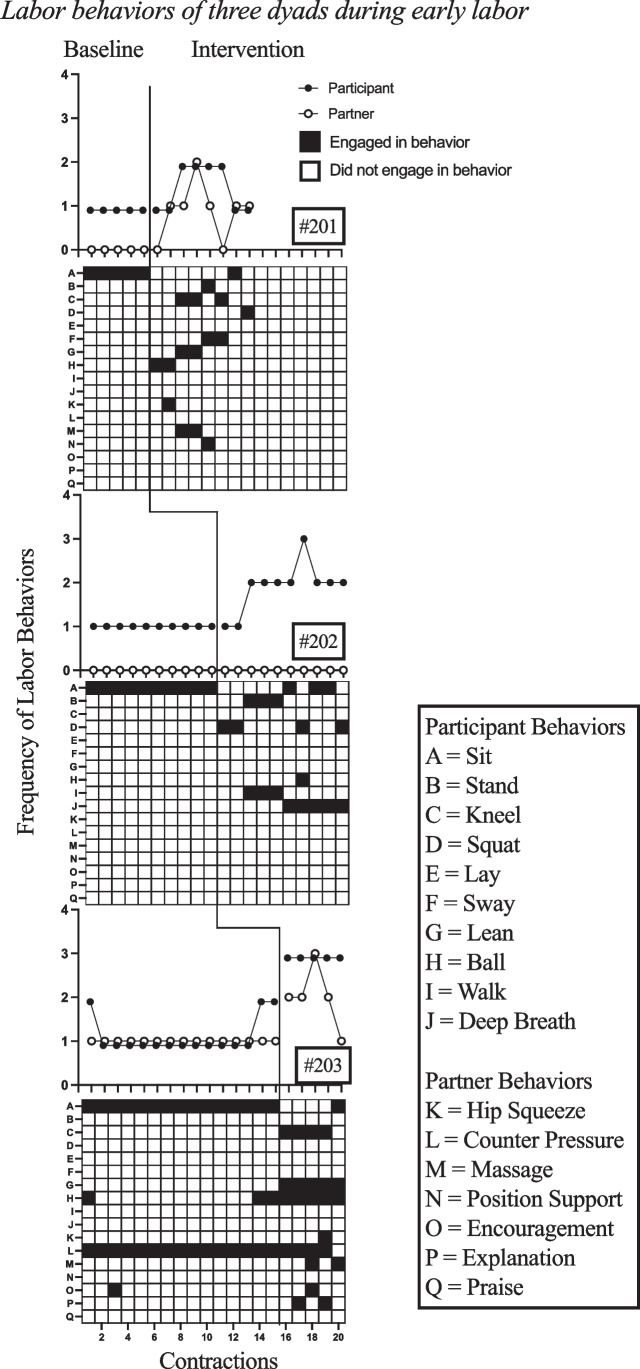
Labor Behaviors of Three Dyads during Early Labor

**Fig. 3:**
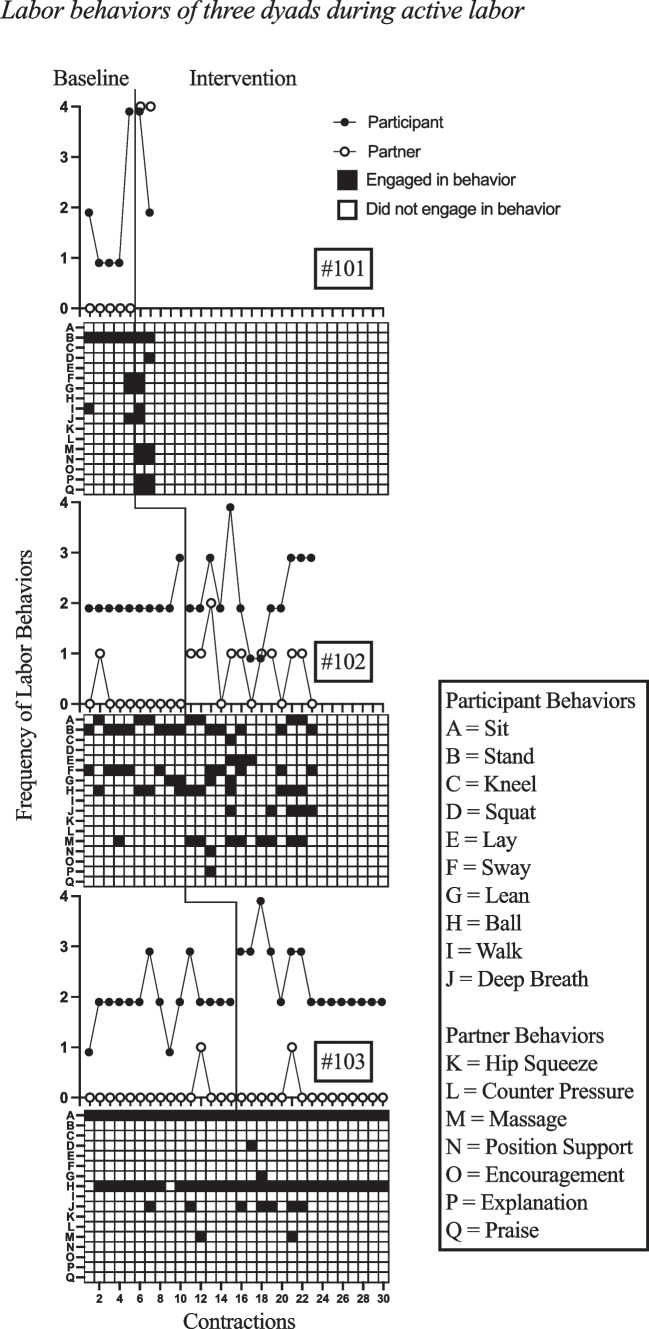
Labor Behaviors of Three Dyads during Active Labor

For ease of distinction between the laboring person and their partner, laboring persons will herein be referred to as the participant (e.g., Participant #201) and the partner will be referred to as such (e.g., Partner #201). Figures 2 and 3 depict responding across baseline and intervention for three dyads. The scatterplot beneath each graph displays the precise labor behaviors engaged in by each participant and partner during each contraction (herein referred to as interval) across baseline and intervention.

The first group of dyads consisted of three participants in early labor with one identified birth partner each. Overall, the dyads’ frequency of labor behaviors increased during the intervention condition (see Fig. 2). Participant #201 consistently engaged in one labor behavior during all baseline intervals. Following the onset of intervention, they had a range of one to two labor behaviors with a mean of 1.5 labor behaviors per interval. Participant #202 engaged in one labor behavior consistently across contractions during baseline and following intervention they engaged in one to three labor behaviors per interval with a mean of 1.9 labor behaviors. Participant #203 engaged in one to two labor behaviors per interval during the baseline condition with a mean of 1.2 labor behaviors per interval, and following intervention, engaged consistently in three labor behaviors per interval, with a mean of 3.0. The partners in the first group showed similar results in that the frequency of labor behaviors during the intervention condition was higher than that of the baseline condition for two of the three participants (see Fig. 2*)*. Partner #201 engaged in zero labor behaviors during baseline, whereas during intervention conditions they engaged in zero to two labor behaviors with a mean of 0.88 labor behaviors per interval. Partner #202 did not engage in any labor behaviors during the baseline or intervention conditions. Partner #203 engaged consistently in one labor behavior throughout the baseline condition and engaged in a range of one to three labor behaviors during intervention, with a mean of two labor behaviors per interval.

In addition to frequency of labor behaviors, variability in labor behaviors was both calculated as described above and although not specifically graphed in this publication, can be gaged through visual inspection of the change in behaviors on the scatterplot of specific behaviors in Fig. 2. Participant #201 did not have any variability in responding during baseline conditions, whereas during intervention they engaged in zero to two novel behaviors per interval, with a mean of 0.88 novel behaviors per interval. Participant #202 also engaged in zero variability in responding during baseline however, they engaged in zero to two novel behaviors per interval during intervention with a mean frequency of 0.9 novel behaviors per interval. Participant #203 engaged in only one novel behavior during baseline, with mean frequency of novel behaviors being 0.07, whereas during intervention they engaged in zero to two novel behaviors per interval with a mean variability of 0.75 per interval. Partner #201 engaged in zero novel behaviors during baseline and between zero to one novel behaviors during the intervention condition, with a mean frequency of novel behaviors of 0.5. Partner #202 did not engage in novel behaviors during baseline or intervention. Partner #203 engaged in one novel behavior during baseline with a mean frequency 0.07 and engaged in zero and two novel behaviors per interval during intervention with a mean frequency of one per interval.

The second group of dyads consisted of a participant and a designated birth partner during active labor. The participants in this group also all demonstrated an increase in overall frequency of labor behavior following the baseline condition (see Fig. 3). Participant #101 engaged in one to four labor behaviors during baseline with a mean of 1.8 behaviors per interval and following intervention engaged in two to four labor behaviors with a mean of three labor behaviors. This participant’s intervention condition was ended prior to the completion of the 2 h time interval because of labor progressing too quickly to complete the entire session. Participant #102 engaged in two to three labor behaviors per interval during baseline with a mean of 2.1 behaviors, whereas they engaged in one to four labor behaviors per interval during intervention with a mean of 2.31 behaviors per interval. Participant #103 engaged in one to three labor behaviors per interval during baseline with a mean of two behaviors and engaged in two to four labor behaviors per interval during intervention with a mean of 2.47 behaviors. The partners’ frequency of labor behaviors increased for two of three participants and remained the same for one participant (see Fig. 3). Partner #101 engaged in zero labor behaviors consistently during the baseline session and engaged in four labor behaviors consistently during intervention intervals. Partner #102 engaged in zero to one labor behaviors during baseline with a mean of 0.1 behaviors per interval and engaged in zero to two labor behaviors per interval during intervention with a mean of 0.77 behaviors per interval. Partner #103 engaged in zero to one labor behaviors during both baseline and intervention conditions with a mean of 0.07 behaviors per interval in both conditions.

The variability for the dyads in active labor demonstrates increases in frequency of novel behaviors for two of the three participants and two of three partners (see Fig. 3). Participant #101 engaged in zero to three novel behaviors during baseline with a mean of 0.75, whereas they engaged in zero to one novel behaviors during intervention with a mean of 0.5. Participant #102 engaged in zero to two novel behaviors during baseline with a mean of 1.11 per interval and they engaged in zero to four novel behaviors with a mean frequency of 1.46 novel behaviors per interval during intervention. Participant #103 engaged in zero to one novel behaviors per interval during baseline with a mean of 0.29 and zero to two novel behaviors with a mean of 0.4 during intervention. Partner #101 did not demonstrate any variability in responding during baseline, however they engaged in zero to four novel responses per interval during intervention, with a mean frequency of two. Partner #102 engaged in zero to one novel behaviors during baseline with a mean of 0.11, whereas they engaged in zero to two novel behaviors per interval during intervention with a mean frequency of 0.46. Partner #103 engaged in zero to one novel behaviors during both baseline and intervention with a mean of 0.07 in each condition.

Data on birth outcomes were also collected as a part of this study (see Table 1). Five of six participants completed the postdelivery survey. Of responding participants, three indicated that they had an unmedicated childbirth, two reported using epidural analgesia during labor, one reported using both intravenous and epidural analgesia during labor, one reported having a cesarean delivery, and four reported having a normal vaginal delivery. Data on birth outcome are intentionally aggregated to protect the participant’s sensitive health information.

**Table 1:** Birth Outcome Data

Measure	Yes		No		Unknown	
	*n*	%	*n*	%	*n*	%
Unmedicated childbirth	3	50.00	2	33.33	1	16.67
Epidural analgesia	2	33.33	3	50.00	1	16.67
IV pain medication	1	16.67	4	66.67	1	16.67
Cesarean section	1	16.67	4	66.67	1	16.67
Assisted vaginal delivery	0	0.00	5	83.33	1	16.67
Normal vaginal delivery	4	66.67	1	16.67	1	16.67

Social validity data were measured through request to continue using the program following the experimental condition and via an exit survey conducted within 4 weeks after the birth. Five of six dyads (all except for Dyad #101) requested to continue using the software program following the experimental condition and they were granted access to keep the iPad with the experimental software for use during the remainder of their labor. In addition, social validity questions were asked on an exit survey (see Table 2). Of responding participants, all five replied yes to the question regarding a positive experience using the software program, three of the partners responded yes, indicating that they were satisfied in using the program, three participants responded yes to a desire to use the software for a subsequent birth, and all five responded yes, that they would recommend the program to a friend.

**Table 2:** Social Validity Survey Outcome Data

Measure	Yes		No		Unknown	
	*n*	%	*n*	%	*n*	%
Laboring person satisfied with StorkAssist	5	83.33	0	0.00	1	16.67
Partner satisfied with StorkAssist	3	50.00	2	33.33	1	16.67
Desire to use it again	3	50.00	2	33.33	1	16.67
Recommend to a friend	5	83.33	0	0.00	1	16.67

## Discussion

The results of the study indicate that the introduction of the software StorkAssist, which includes immediate prompting and modeling of behavioral coping strategies, encouraged an imitative repertoire during labor, thereby increasing the use of such strategies during early and active labor.

Active engagement during childbirth encourages the natural progression of labor as posture and positioning of the birthing person aids in optimal fetal positioning, allowing the baby to settle into the person’s unique pelvis in an ideal position for birth (Odneck, 2014). For example, walking during labor leverages gravity and helps move and open the pelvis, allowing the baby to both rotate and descend. If the baby is in an optimal position in the pelvis, the birth process will be quicker and the use of cesarean section or other major interventions to assist with delivery will be less likely to be necessary (Chaillet et al., 2014). In addition to helping the baby settle into the pelvis, movement and positioning help the birthing person cope with the pain and other challenges associated with labor (Odneck, 2014).

The current participants and their partners increased the frequency and variability of their labor behaviors during the intervention condition. During baseline for the early labor dyads, partners were largely uninvolved and did not engage in many labor specific behaviors whereas the laboring persons in the early labor group mostly sat in one position without much other movement. During the intervention condition, all three early labor dyads began to engage in higher frequencies of labor behaviors. Although there was a less noticeable effect for the active labor dyads, the range and mean frequencies of labor behaviors did increase during the intervention condition for all three dyads. The three participants engaged in some labor behavior during baseline but engaged in higher frequencies following intervention. The three partners engaged in little labor behavior during baseline, but following intervention, two of three partners engaged in higher frequencies of labor behavior.

Variability of behavior during labor is also important as positional changes by the laboring person also help the baby settle into the pelvis in optimal fetal positioning, allowing for ease of birth (Odneck, 2014). In a study examining the various factors that contribute to a normal physiological birth, freedom of movement throughout labor was identified as a main variable in in promoting birth without assisted delivery or cesarean section (Prosser et al., 2018). In addition, novel coping strategies may be helpful for both the laboring person and their partner to try as the various physical and emotional challenges of childbirth arise. In examining the specific labor behaviors of the dyads, new behaviors were attempted and several of these behaviors were repeated or sustained throughout the intervention condition, likely indicating that the participants and their partners contacted reinforcing effects of these new behaviors.

The social validity survey demonstrates that the laboring persons felt satisfied with their experience using StorkAssist and that they would recommend it to a friend. The requests to keep the iPad following the experimental condition is also suggestive that there were some components of the treatment package that participants found valuable. Anecdotal comments by the participants collected in the postdelivery social validity survey also indicate that the software was beneficial to the participants and the partners during labor:

We just wanted to say thank you for having us participate in your study, the app was super helpful especially when we started running on fumes! The reminders and different positions really helped when something stopped working and we needed to change things up. Once we got past a certain point, we didn't use the app anymore, but the things we were able to glean from using it in the beginning came back to us while we were in the major throws of things.

It is worth noting that there was a lower desire to use StorkAssist again, even though most reported it beneficial. This is likely the result of StorkAssist being a pilot demonstration software that could be further developed to improve user experience and overall satisfaction and desire to use it again.

The availability and use of the StorkAssist application likely increased the occurrence of and variability of labor behaviors. In particular, almost all the participants remarked in the social validity survey that they would not have remembered what they had learned in childbirth education classes if not for the immediate prompts provided by StorkAssist. As behavior analysts, this is not surprising as the generalizability of one trial learning that occurs during childbirth education is ineffective. One participant specifically commented, “When I am laboring, I forget what could be effective. I was much more open to trying different positions this birth. I think the program helped because I was able to get ideas instantly.” Another remarked, “It's so helpful to have all of the position options available. After learning them in a class or reading about them, it would have been difficult to recall them all on our own, not to mention which would be right to use when.” A third said, “It suggested positions that we had not thought of. Even though I had read about all the positions before, at the time of labor I wasn't thinking of them. It helped remind me and get me into different positions to see what was working.”

From a behavioral perspective, what was observed to occur during the use of StorkAssist is imitation resulting from a video model that serves as an antecedent stimulus, evoking the imitative response. The visual prompts of the coping strategies serve as an opportunity for choice selection, whereas the video model evokes the imitative behavior, consistent with the definition of imitation (Holth, 2003) in which the behavior must immediately follow the presentation of the model, have formal similarity, and the model must be the controlling variable of the imitative behavior. The temporal nature of the demonstration and the immediacy of the engagement in the behavior following the model is likely why this treatment package is so successful, whereas classroom learning of coping skills, even if practiced to fluency, does not generalize into this novel environment and situation.

Partners have a responsibility during labor to provide support and comfort, and 30 years of research supports that people have better birth outcomes with good partner support (Green & Hotelling, 2014). Research demonstrates that appropriate and adequate labor support reduces birth trauma and likelihood of postpartum depression (Hodnett et al., 2012). A review of the literature conducted by Van der Gucht and Lewis (2015) concluded that continuous and individualized support was a contributing factor to positive birth outcomes for laboring persons, regardless of cultural differences. In addition, the availability of a low-cost intervention such as the StorkAssist, may be beneficial to laboring persons and/or partners who were unable to access childbirth education or those without specific knowledge of coping strategies.

There are several good reasons to encourage movement and position changes during labor. Studies have concluded that people prefer to be in upright positions during labor and have a greater sense of control (i.e., Gilder et al., 2002). Research also demonstrates that mobility during labor reduces duration of labor and has implications for improved birth outcome (Lawrence et al., 2013). People are told to change positions every 30–60 min while in labor, however an exact time for position changes during labor is not formally documented. This technology could be adopted in hospital, birth center, or home settings to encourage laboring persons to try different positions, could be adapted to include time prompts for position changes or restrictions on playing the same video model for more than a set period. A highlight of StorkAssist is the ability to use this software without any pretraining or knowledge, therefore making it easy to incorporate into any birth setting.

As seen in the data, the laboring participants in early labor engaged with StorkAssist more than those in active labor, indicating that as the intensity of contractions increased, there was a reduction in the use of the software from the laboring person. The exact reason for this is unknown, however, it can be assumed that as labor intensity increases, it is harder to engage with external stimuli, because many people instinctively begin to close their eyes as contraction intensity increases. It is also possible that the imitative repertoire of and formal similarity to the model became automatically reinforcing through reduction of painful stimulation, therefore, the laboring persons didn’t need or want to engage with the software as frequently once they began to find what was most comfortable to them. It is important to note, however, that as labor progressed, there was not a noticeable difference in partner support behavior, indicating that StorkAssist may be beneficial for partners during early and active labor.

There are several limitations to this study. First and foremost, although data on birth outcomes were collected and reported, due to the small sample size and the inability to carry the experimental condition into the hospital setting for the duration of labor, correlation between the intervention and birth outcomes cannot be made. However, the preliminary outcome data do suggest that analgesia use is less than half of the national average and cesarean section rate is a third less than that of the national average for the six dyads that participated. In addition, there are medical and social variables that could not be controlled for, including the care provider delivering the baby, nursing staff responsible for patient care, preferences and desires of the participants, and number of childbirth education hours/self-study that participants engaged in. Another limitation is that the nature of the research likely attracted participants that were hoping for a more natural approach to childbirth. Finally, the progression and intensity of labor itself cannot be controlled for, something that may have influenced participant responding.

Future researchers on this topic might focus on the replication and extension of this study to include a larger population size as well as a control group. More rigorous methodologies might also be included such as use of an electronic contraction monitor to determine the onset and conclusion of each contraction more precisely. In addition, research might focus on identifying certain variables of the treatment package that were most effective. Finally, future research might include examining the entire duration of labor within the experimental condition to better determine the relationship between intervention and birth outcome.

The purpose of this study was to examine how a software treatment package incorporating prompting, choice, and modeling of labor behaviors effected the frequency and variability of labor behaviors engaged in by birthing persons and their partners. The packaged intervention proved to be useful to engage with and was easily incorporated into the labor experience of each dyad. The StorkAssist program appears to have successfully promoted imitation of coping skills and an increase in the frequency and variability of labor behaviors for participants and their partners during unmedicated early and active labor. All five dyads reported favorable outcomes and satisfaction with their experience using the software, reporting it to be beneficial during their labors. With decades of research discussing the need for more effective prompting methodologies of coping strategies during childbirth, the results of this study are promising and suggest that a behavior analytic technology applied directly to childbirth can increase labor behaviors, and hence, coping and partner support strategies, with no prior training required.

## Data Availability

The datasets generated during and/or analyzed during the current study are available from the corresponding author on request.

## Footnotes

1 Providing a comprehensive list of all options included in StorkAssist is prohibitive but for additional information, please contact the author directly.

2 At the time that this this study was conducted, the researcher and the research assistant were both trained doulas with experience in childbirth.
